# Significant Decrease in Hippocampus and Amygdala Mean Diffusivity in Treatment-Resistant Depression Patients Who Respond to Electroconvulsive Therapy

**DOI:** 10.3389/fpsyt.2019.00694

**Published:** 2019-09-19

**Authors:** Antoine Yrondi, Federico Nemmi, Sophie Billoux, Aurélie Giron, Marie Sporer, Simon Taib, Juliette Salles, Damien Pierre, Claire Thalamas, Laurent Schmitt, Patrice Péran, Christophe Arbus

**Affiliations:** ^1^Service de Psychiatrie et de Psychologie Médicale, Centre Expert Dépression Résistante FondaMental, CHU Toulouse, Hospital Purpan, ToNIC, Toulouse NeuroImaging Center, Université de Toulouse, Inserm, UPS, Toulouse, France; ^2^ToNIC, Toulouse NeuroImaging Center, University of Toulouse, Inserm, UPS, Toulouse, France; ^3^Service de médicine légale, CHU Toulouse, Toulouse, France; ^4^Service de Psychiatrie et de Psychologie Médicale, CHU de Toulouse, Hospital Purpan, Toulouse, France; ^5^Service de Psychiatrie et de Psychologie Médicale, CHU Toulouse, Hospital Purpan, ToNIC, Toulouse NeuroImaging Center, Université de Toulouse, Inserm, UPS, Toulouse, France; ^6^Service de Psychiatrie et de Psychologie Médicale, Centre Expert Dépression Résistante FondaMental, CHU Toulouse, Hospital Purpan, Toulouse, France; ^7^CIC 1436, Service de Pharmacologie Clinique, CHU de Toulouse, INSERM, Université de Toulouse, UPS, Toulouse, France

**Keywords:** depressive disorder, electroconvulsive therapy, MRI, DTI, mean diffusivity

## Abstract

**Introduction:** The hippocampus plays a key role in depressive disorder, and the amygdala is involved in depressive disorder through the key role that it plays in emotional regulation. Electroconvulsive therapy (ECT) may alter the microstructure of these two regions. Since mean diffusivity (MD), is known to be an indirect marker of microstructural integrity and can be derived from diffusion tensor imaging (DTI) scans, we aim to test the hypothesis that treatment-resistant depression (TRD) patients undergoing bilateral (BL) ECT exhibit a decrease of MD in their hippocampus and amygdala.

**Methods:** Patients, between 50 and 70 years of age, diagnosed with TRD were recruited from the University Hospital of Toulouse and assessed clinically (Hamilton Depression Rating Scale, HAM-D) and by DTI scans at three time points: baseline, V2 (during treatment), and V3 within 1 week of completing ECT.

**Results:** We included 15 patients, who were all responders. The left and right hippocampi and the left amygdala showed a significant decrease in MD at V3, compared to baseline [respectively: β = −2.78, t = −1.97, p = 0.04; β = −2.56, t = −2, p = 0.04; β = −2.5, t = −2.3, p = 0.04, false discovery rate (FDR) corrected]. MD did not decrease in the right amygdala. Only the left amygdala was significantly associated with a reduction in HAM-D (ρ = 0.55, p = 0.049, FDR corrected).

**Conclusion:** MD is an indirect microstructural integrity marker, which decreases in the hippocampus and the left amygdala, during BL ECT in TRD populations. This could be interpreted as a normalization of microstructural integrity in these structures.

## Introduction

With more than 300 million people affected and a high lifetime prevalence of 16% to 17% in the general population (1), major depressive disorder (MDD) is an increasingly widespread illness. Treatment-resistant depression (TRD) has been estimated to represent half of the overall treatment costs for major depression (2). It can be assumed that approximately 20% to 30% of depressed patients experience TRD (3), with up to one-half of depressed patients not achieving remission (responding only partially) (3). TRD is currently defined as the failure of at least two rounds of antidepressant treatments, administered sequentially and at the appropriate dose and duration (4). TRD is known to have a heterogeneous etiology (4). Some sources of variability include different environmental risk factors (e.g., childhood adversities), multiple genetic determinants (related to numerous genetic loci), and various epigenetic contributors (e.g., hypothalamic–pituitary–adrenal axis, immune function, monoamines, neurotrophic factors, etc.) (2, 5–8). Despite this heterogeneity, electroconvulsive therapy (ECT) remains the treatment of choice for severe TRD (9).

However, the exact mechanism of ECT remains unclear (10). There are many hypotheses that can explain its efficacy, albeit only in part: involvement of the (i) monoamine pathway [for details, see Ref. (11)], (ii) immune inflammation [for details, see Ref. (12)], and (iii) neuroplastic changes [for details, see Ref. (13)]. Currently, most structural neuroimaging studies focus on changes in hippocampus and amygdala volumes after ECT (14). Indeed, the hippocampus plays a key role in depressive disorder, and the amygdala is involved in depressive disorder through the key role that it plays in emotional regulation (15, 16). Moreover, many studies show an increase of hippocampus and amygdala volume after treatment (14, 17–19). Only two studies focus on microstructural changes in the hippocampus and amygdala (20, 21). Jorgensen et al. (20) show a decrease of mean diffusivity (MD) in the hippocampus between baseline and the end of treatment in a heterogeneous population (unipolar and bipolar disorder). Kubicki et al. (21) also show a decrease in MD in the right hippocampus in a TRD population using a right unilateral (RUL) ECT. None of these studies evaluated the change in MD during the course of treatment. MD, derived from diffusion tensor imaging (DTI) scans, is nevertheless known to be an indirect marker of microstructural integrity (22). It is therefore conceivable that ECT could have a microstructural effect on brain structures (specifically on the hippocampus and the amygdala). We set out to test this hypothesis by assessing whether a decrease in MD could be detected in the hippocampus and the amygdala in TRD populations during bilateral (BL) ECT. Indeed, the decrease of MD in the hippocampus and the amygdala could be an additional argument to support the thesis that the increases in hippocampal (17, 18) and amygdala (17) volumes are not merely due to localized edema, which would be expected to increase water diffusivity. Our main objective is to assess whether there is a decrease of MD in the hippocampus and amygdala in TRD populations during ECT (bitemporal stimulation). Our secondary objective is to evaluate whether this decrease is associated with a clinical improvement.

## Methods

### Participants

Patients scheduled to begin ECT were recruited from the University Hospital of Toulouse. Eligibility criteria included a diagnosis of TRD (Thase and Rush > = 2) (23) and patient age between 50 and 70 years. We chose to restrict the population of the present study to subjects between 50 and 70 years of age, to select a more homogeneous population in terms of aging brain structures. Patient diagnoses were established by clinical consultation using the *Diagnostic and Statistical Manual of Mental Disorders 5* (*DSM 5*) (24) criteria. Patients with independently diagnosed co-morbid psychiatric disorders, including schizophrenia, schizoaffective disorders, bipolar disorder, post-traumatic stress disorder, attention deficit/hyperactivity disorder, and dissociative disorders, and patients independently diagnosed with anxiety disorders were excluded from the study. Other exclusion criteria included a co-morbidity of dementia, traumatic brain injury (TBI), depression related to a medical condition, ECT or other neuromodulatory therapies in the previous 6 months, or ECT and anesthesia contraindications. Additional exclusion criteria included a history of alcohol or substance abuse within 6 months and/or dependence within 12 months of participation, neurological disorders, and conditions contraindicating magnetic resonance imaging (MRI). Antidepressant treatment was no longer modified after the patient’s inclusion into the study (**Table 1**).

**Table 1:** Antidepressive pharmacological treatment.

Case No	Antidepressant (mg/d)	Add on (mg/d)
1	Amit (125)	
2	Amit (100)	
3	Clom (150)	
4	Vort (20)	
5	Amit (175)	
6	Clom (225)	
7	Fluo (60) ; Mian (30)	
8	Sert (200)	
9	Venl (300)	
10	Clom (150)	
11	Clom (150) ; Mirt (30)	Lam (200)
12	Esci (30)	
13	Amit (100)	
14	Fluo (20)	
15	Venl (75) ; Mirt (45)	

No, number; Mg/d, milligram per day; Amit, Amitriptyline; Clom, Clomipramine; Vort, Vortioxetine; Fluo, Fluoxetine; Mian, Mianserine; Sert, Sertraline; Venl, Venlafaxine; Esci, Escitalopram; Mirt, Mirtazapine; Lam, Lamotrigine.

Of a total of 17 patients enrolled, we were able to collect data for all three time points for 15 (6 females and 9 males) (**Table 2**). Of the two patients who were lost to the study, one continued ECT without MRI assessment, and one discontinued ECT early. This is an exploratory study. A sample size of 15 subjects is lower than the number required in this type of study, which is normally set at 30. Indeed, estimates determined by MRI are expected to be more reproducible than those obtained from other types of measurements (i.e., biological, psychometric) when using an region of interest (ROI) approach, since the MRI data are averaged over a given area. The inclusion of 15 patients will therefore be sufficient to meet the target. In addition, the Nordanskog study (25) estimates an average 255 mm^3^ increase in total hippocampus volume (but not dispersion) after a first session of ECT, as reported in their study of 12 severely depressive subjects, with a mean age of 40 years. By analyzing 15 patients, the current study has a 90% power of detecting significant differences, with a standard deviation of up to 272 mm^3^, using t-tests for series matched with a fixed alpha risk of 0.05.

**Table 2:** Demographic and clinical data.

	Patients (SD)
Gender (Female/Male)	6/9
Age	59.2 (7.1)
Education (years)	11.57 (3.27)
HAM-D Baseline (0-52)	22.8 (3.05)
HAM-D endpoint (0-52	4 (2.65)
Suicide attempt (number)	1.33 (1.99)
Number of hospitalization (for MDD)	2.13 (1.73)
Thase and Rush staging (1-5)	2.4 (.63)
ECT (No)	12.1 (5.26)

ECT, electroconvulsive therapy; HAM-D, Hamilton Depressive Rating Scale; MDD, major depressive disorder; No, number; SD, standard deviation.

All participants provided written informed consent for participation as approved by the Comité de Protection des Personnes (CPP) Sud Ouest Outre Mer 4 (CPP15-053) (i.e., the fourth ethics research committee of southwestern France and French overseas territories). This study was carried out in accordance with the recommendations of the CPP Sud Ouest Outre Mer 4 (CPP15-053). The protocol was approved by the CPP Sud Ouest Outre Mer 4 (CPP15-053).

### Data Acquisition

Patients were assessed clinically and by structural scans at three time points: V1 (baseline) completed within 48 h before the first ECT; V2 completed within the first ECT that was considered effective (as defined by the RCPsych in the *ECT Handbook*: effective ECT seizures should consist of a convulsion lasting 15 s or more or of an EEG recording showing seizure activity lasting 25 s or more) (26); and V3 performed within 1 week of completing ECT. For 5 patients, the first ECT considered effective was the first treatment; for the remaining 10, it was the second treatment.

### Clinical Assessment

The 17-item Hamilton Depression Rating Scale (HAM-D) (27) was administered at each time point to assess symptoms and treatment responses: before the first treatment session, after the first ECT that was considered effective (same day), and 1 week after the last ECT session. Individual patients were assessed by the same evaluator at the three time points.

### ECT

ECT (5000Q MECTA, Tualatin, OR, USA) was administered twice a week, using a standard protocol for anesthesia (propofol) and paralysis (succinylcholine). ECT followed the seizure threshold (ST) titration method, where following establishment of the ST, treatments are delivered at 1.5× to 2× ST for BL (using brief pulse width). The length of ECT is determined on an individual clinical basis. Patients received a mean of 12.07 ECTs (**Table 2**).

### MRI

A brain MRI was performed for all participants using a 3T MR imager (Intera Achieva, Philips, Best, Netherlands) with a 32-channel head coil at the Inserm/UPS UMR1214 ToNIC Technical Platform, Toulouse, France. We acquired a three-dimensional (3-D) T1-weighted sequence using a gradient echo (170 sagittal slices; scan mode: 3-D; multishot; contrast T1; voxel resolution (mm^3^): 1.00 × 1.00 × 1.00; acquisition time: 10’14”).

Diffusion-weighted volume measurements were acquired using a spin-echo echo-planar imaging (EPI) (95 slices; voxel size 1.5 × 1.5 × 1.5 mm^3^) with 32 isotropically distributed orientations for the diffusion-sensitizing gradients at a b-value of 1,000 s/mm² and a b = 0 images (acquisition time: 16’09”).

Structural images were processed using FreeSurfer’s image analysis pipeline (version 5.3.0). To extract reliable volume, the images were automatically processed with the longitudinal pipeline in FreeSurfer (28).

From the diffusion images, the MD (FMRIB’s Diffusion Toolbox, FSLv5) was calculated. The image acquired at b0 was co-registered on the T1 image through an affine transformation (cost function: standardized mutual information), and the transformation matrix was then applied to MD maps. The enabled the depiction of T1 and MD images in the same space, the individual space.

### Statistical Analysis

#### Analyzing HAM-D at the Different Time Points

To test the association between ECT sessions and HAM-D values, we fitted a mixed linear model with HAM-D values as a dependent variable and time points (coded as a continuous variable) as an independent variable. We included a random intercept for each subject in the model. Models were fitted using the “nlme” package (29) in R (https://www.r-project.org/).

#### Analyzing MD at the Different Time Points

To test the association between ECT sessions and MD in the amygdala and hippocampus, we fitted different mixed linear models for each structure and hemisphere. We entered the average MD values for the structures of interest as a dependent variable and the time point (coded as a factor) and the sex and age of the patients as independent variables. We included a random intercept and slope for each subject in the model. In light of previous findings (21), we hypothesized that we would observe a decrease in MD with time. As such, we calculated one-tailed (i.e., β lower than zero) p values. These values were corrected for FDR. Models were fitted using the “nlme” package (29) in R (https://www.r-project.org/).

#### Analyzing the Association Between MD and HAM-D Decrease

To test possible associations between MD and HAM-D decrease, we ran correlations between delta HAM-D score (V3-T1) and delta MD in selected sub regions (V3-T1). We then performed a Spearman non-parametric correlation between “HAM-D delta” and “MD delta” separately for the relevant structures. Since we were expecting a positive association between the delta values (i.e., the greater the reduction in MD, the greater the decrease in HAM-D), we calculated one-tailed p values.

## Results

### Demographic Data

The demographic data of the 15 patients enrolled in the study are summarized in **Table 2**.

### Clinical Data

Using a threshold of a 50% improvement in symptoms at the end of the treatment index, 100% (n = 15) of patients are categorized as responsive to treatment. Using a threshold of HAM-D < = 7, 93.3% (14 patients) went into remission.

### HAM-D and Time Point

Relative to V1, there was a significant decrease in HAM-D both at V2 (β = − 2, t = −2.62, p < 0.000) and at V3 (β = −18.8, t = −16.44, p < 0.000).

### MD and Time Point

**Figure 1:**
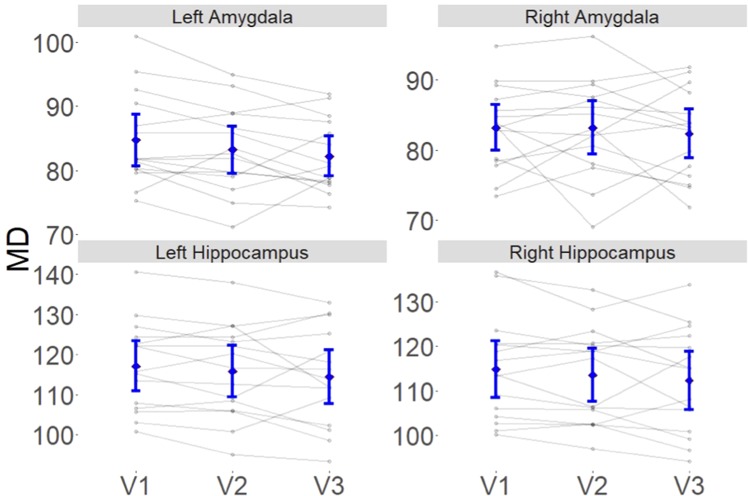
Single subjects measures and trajectories (in black) and group average and 95% CI (in blue) for V1, V2 and V3. In the left and right hippocampus, and the left amydala, the average MD was significantly lower at V3 than V1 (p = 0.4). Note that MD has been scaled by multiplying its original value by 1000.

- Left hippocampus: relative to V1, the MD was unchanged at V2 (β = −1.33, t = −.97, p = 0.17), but there was a decrease in MD at V3 (β = −2.78, t = −1.97, p = 0.04).
- Right hippocampus: relative to V1, the MD was unchanged at V2 (β = −1.31, t = −1.03, p = 0.15), but there was a decrease in MD at V3 (β = −2.56, t = −2, p = 0.04).
- Left amygdala: relative to V1, the MD was unchanged at V2 (β = −1.5, t = −1.53, p = 0.067), but there was a decrease in MD at V3 (β = −2.5, t = −2.3, p = 0.04).
- Right amygdala: there was no decrease in MD relative to V1, V2 (p = 0.5), or V3 (p = 0.25).
- (**Figure 1**.)

### FA and Time Points

We observed no differences between time points in the selected ROIs using fractional anisotropy (FA) as dependent variables (all p values > 0.43 FDR corrected and >0.18 when not corrected).

### Relationship Between MD and HAM-D Decrease

Of the three structures that showed a significant decrease in MD, only the left amygdala showed an association with a reduction in HAM-D (ρ = 0.55, p = 0.017). The association between delta HAM-D and delta MD was not significant for the left (p = 0.35) and right (p = 0.3) hippocampus.

## Discussion

To our knowledge, this is the first study assessing a change in MD in the hippocampus and amygdala during BL ECT in a TRD population.

MD could be considered as a marker of the overall displacement of water molecules. Increased values are hypothesized to reflect degeneration of cellular membranes or inflammation and may consequently provide a more sensitive indication of age-related brain changes (30–32) or of the effects of various brain pathologies, such as depressive disorders (33). Focusing on MDD, MD changes appear as soon as the subclinical symptoms appear (34). Moreover, Pal et al. show an age-related decrease of MD in grey matter with age in the healthy population, but no change was associated with gender (35). Due to its small age range, our sample minimizes any confounding age-associated effects. The anterior cingulate cortex, amygdala, and hippocampus also form part of an interconnected prefrontal neocortical and limbic archicortical network that is dysregulated in MDD (15, 16), which is why numerous studies have focused on these brain structures in depressive disorders. The decrease of hippocampal MD seems to be associated with subclinical symptoms and appears very early in the pathology (34). Moreover, many studies report an increase of hippocampus (17, 18) and amygdala (17) volumes during ECT. Taking account of these findings reported in the literature, we therefore focused on the hippocampus and amygdala, even though other brain structures, such as the anterior cingulate cortex, prefrontal cortex, etc., may also be of interest.

Assessing differences in hemispheric asymmetry is difficult, as such differences exist not only in the healthy population (36–38) but also in MDD patients (39). Jiang et al. indeed highlight that similar patterns are observed between functional and structural networks: overall, the right hemisphere is over-connected and more efficient than the left hemisphere; the occipital and parietal regions exhibit leftward asymmetry; and the frontal and temporal sites show local rightward lateralization with regard to regional connectivity profiles. The Jiang et al. study also shows that the functional–structural coupling of intra-hemispheric connections is significantly decreased and correlated to disease severity (39). However, to our knowledge, there are no available data focusing on hemispheric asymmetry in relation to MD of the hippocampus and amygdala in an MDD population. Nonetheless, Madsen et al. highlight that higher left relative to right hippocampus MD is associated with higher basal cortisol levels in the healthy population (40). It is also known that basal cortisol levels may be raised in MDD (41).

In the present study, we showed a decrease in MD in the left hippocampus, right hippocampus, and left amygdala during treatment. But we did not find a decrease in the right amygdala. Lorenzetti et al. reported an amygdala asymmetry in MDD populations in remission. They found that left amygdala volumes of subjects in remission were significantly larger when compared to healthy controls and tended to be larger when compared to subjects who were not in remission. In contrast, the Lorenzetti study did not observe any differences in left amygdala volumes between MDD patients and healthy controls. In addition to this, right amygdala volumes do not differ between the groups (42). Moreover, Chen et al. highlight that variations in neural activity are greater in the left amygdala than the right following antidepressant treatment (43). These data could, at least partially, explain the difference in MD decreases we observed in the amygdala.

Moreover, we show an association between MD decrease and HAM-D reduction in the left amygdala.

We did not observe any effect of ECT session on the FA of the selected ROIs. This result is not surprising: FA is an index known to be related to fiber orientation, as such, it is less sensitive than MD to microstructural changes in the grey matter. If anything, our results confirm that MD is a better DTI-related index than FA for testing grey matter microstructure *in vivo*.

As regards MD, our results are consistent with two previous studies (20, 21) that focused on MD but used a different technique (RUL ECT) (21) and analyzed a more heterogeneous and non-resistant population (20). Unlike Kubicki et al. (21), we do not show any association between a decrease in MD in hippocampus and a decrease in HAM-D. Unlike Kubicki et al. (21), we show an association between an MD decrease and a HAM-D reduction in the left amygdala.

Our results, in addition to these two other studies (20, 21), could be supportive of a neurotrophic and neuroplasticity hypothesis. Indeed, MD is known to be an indirect marker of microstructural integrity. For example, in Alzheimer’s disease, Gerischer et al. (22) show that MD is significantly increased in the patient group, indicating a loss of integrity of tissue microstructure, with significantly smaller hippocampal volumes. The decrease of MD during ECT could be interpreted as a normalization of microstructural integrity in these structures. This reduction in hippocampal MD seems to indicate an increase in hippocampal volume (18) that is not merely attributed to localized edema but could also be expected to lead to an increase in water diffusivity. Some animal studies have highlighted an increase of brain derived neurotrophic factor (BDNF) in the hippocampus (44). Bouckaert et al. (45) show an association between increased hippocampal volume and BDNF concentrations in human serum. Many studies of animal populations highlight that ECT could induce a neurotropic action in the hippocampus: increase of neurogenesis, synaptogenesis, and glial cell proliferation (44, 46). Finally, numerous studies have observed neuroplasticity following ECT in various regions of interest, such as the hippocampus (45, 47).

Nonetheless, we have to take into consideration that as a result of the small number of patients enrolled in our study (15), our population is very narrow. Indeed, although this is a population with severe TRD according to the thresholds recommended in the American Psychiatric Association (APA) *Handbook of Psychiatric Measures* (48) to define grades of severity on the HAM-D [mild to moderate: 8–18; severe: 19–22; very severe ≥23 (range from 19 to 29 in our sample)] and according to the thresholds recommended in the Thase and Rush scale to define grade of resistance [resistance ≥2 (23); (range from 2 to 4 in our sample)], the severity and the resistance could be considered as relatively low, with, in addition to this, two attempted suicides and two lifetime hospitalizations for depressive disorders. Moreover, the patients are rather old (50–70 years). These clinical data could explain the very high rates of response and remission in our sample. All these issues should be taken into account when extrapolating from our results.

## Limitations

Our study has some limitations. First, we only included a small number of patients (15). In light of this, we could not analyze different MDD clusters. Thus, this increases the risk of a heterogeneous population. Moreover, we decided to maintain antidepressant treatment during ECT (as we do in daily care in our unit). This can affect MD changes. But we did attempt to limit this effect by not altering the antidepressant treatment after inclusion into the study. Moreover, all patients responded to the treatment, with 14 out of the 15 patients achieving remission. This introduces a bias, as we are unable to differentiate whether we are assessing ECT mechanisms or an antidepressant effect of ECT. Adding two control groups, one only treated with antidepressants and the other a healthy control group, would allow us to differentiate between these two ECT mechanisms. In addition to this, the female/male ratio in our study is approximately 0.67, whereas the ratio reported in the literature is around 2 (49). So, our results should be interpreted with caution. Finally, we did not take the neurocognitive data into account to interpret our results.

## Strengths

In order to avoid introducing undue heterogeneity caused by differential aging of brain structures, we chose to restrict our study population to patients (i) ranging from 50 to 70 years of age (ii) and diagnosed with severe unipolar TRD. Furthermore, all patients were treated with bitemporal stimulation.

## Perspectives

It seems that there are two main ways to improve our understanding of ECT mechanisms.

Firstly, the immuno-inflammation pathway seems to be interesting. Indeed, Maes et al. (50) suggest that an increase of inflammatory cytokines is associated with a decrease of neurogenesis (with a decrease in BDNF, fibroblast growth factor (FGF), and neural cell adhesion molecule (NCAM)). Moreover, in a previous review (12), we reported that ECT could affect immune response and inflammation. It is interesting to take these data into consideration to identify a link between neuro-inflammation, MD change, and ECT. Currently, in addition to peripheral biomarkers of inflammation such as CRP, interleukin (IL)-6, IL-1 beta, and tumor necrosis factor (TNF)-alpha, we are able to assess microglia activation in MDD as a neuro-inflammatory biomarker using translocator protein (TSPO) density in positron emission tomography (PET) scans (51). In addition to biological and MRI assessment, it could be interesting to use PET scans (TSPO density) to assess the effects of neuro-inflammation on MD changes during ECT.

Secondly, exploring any potential associations between changes in MD and epigenetic effects could be an interesting avenue to gain a better understanding of ECT mechanisms. Indeed, epigenetic regulators can be defined as molecules that directly or indirectly regulate gene expression. Although protein-coding genes and their respective messenger RNAs (mRNAs) are functional effectors of epigenetic differences, how epigenetic changes are associated with MDD pathology and/or influenced by treatment can be investigated to understand underlying pathophysiological changes. DNA methylation and post-translational histone modifications are the most widely accepted epigenetic regulators (52). Some studies have identified epigenetic changes that occur during antidepressant treatment in patients who respond to treatment. Indeed, the expression of some micro RNAs (miRNA) changes in patients who respond to treatment (miRNA 16, miRNA 135a, miRNA 9, miRNA 326, miRNA 1202, miRNA 335, miRNA 24, miRNA 146a and b, and miRNA 425) (52). It will be interesting to assess if MD changes could be due to epigenetic regulators as miRNA expression changes.

Finally, it could be interesting to evaluate whether changes in MD are associated with neurocognitive impairments that can occur during ECT.

## Conclusion

MD, which is known to be an indirect marker of microstructural integrity, decreases in the hippocampus and left amygdala during BL ECT in TRD populations. This could be interpreted as a normalization of microstructural integrity in these structures. These findings could suggest the involvement of a neurotrophic and/or inflammatory process in the ECT mechanism.

## Data Availability

The datasets generated for this study are available on request to the corresponding author.

## Ethics Statement

All participants provided written informed consent for participation as approved by the CPP Sud Ouest Outre Mer 4 (CPP15-053) (i.e., the fourth ethics research committee of southwestern France and French overseas territories). This study was carried out in accordance with the recommendations of the CPP Sud Ouest Outre Mer 4 (CPP15-053). The protocol was approved by the CPP Sud Ouest Outre Mer 4 (CPP15-053).

## Author Contributions

AY: Co-writing protocols, inclusion/recruitment, clinical assessment, neuroimaging analysis, statistical analysis, co-writing paper. FN: Neuroimaging analysis, statistical analysis (neuroimaging), co-writing paper. SB, AG, MS, ST, JS, DP, LS: Inclusion/recruitment, clinical assessment, co-writing paper (clinical part). CT: Co-writing protocols, methodology, statistical analysis (clinical data). CA: Co-writing protocols, grant recipient (principal investigator), co-writing paper. PP: Co-writing protocols, neuroimaging analysis, statistical analysis (neuroimaging), co-writing paper.

## Funding

This work was supported by a grant from the Délégation Régionale à la Recherche Clinique des Hôpitaux de Toulouse “2015.”

## Conflict of Interest Statement

The authors declare that the research was conducted in the absence of any commercial or financial relationships that could be construed as a potential conflict of interest.
